# Is non-high-density lipoprotein associated with metabolic syndrome? A systematic review and meta-analysis

**DOI:** 10.3389/fendo.2022.957136

**Published:** 2022-09-13

**Authors:** Parham Mardi, Fatemeh Abdi, Amir Ehsani, Ehsan Seif, Shirin Djalalinia, Javad Heshmati, Ehsan Shahrestanaki, Armita Mahdavi Gorabi, Mostafa Qorbani

**Affiliations:** ^1^ Non-Communicable Diseases Research Center, Alborz University of Medical Sciences, Karaj, Iran; ^2^ Student Research Committee, Alborz University of Medical Sciences, Karaj, Iran; ^3^ University of Medical Sciences, Tehran, Iran; ^4^ Development of Research and Technology Center, Deputy of Research and Technology, Ministry of Health and Medical Education, Tehran, Iran; ^5^ Non-Communicable Diseases Research Center, Endocrinology and Metabolism Population Sciences Institute, Tehran University of Medical Sciences, Tehran, Iran; ^6^ Songhor Healthcare Center, Kermanshah University of Medical Sciences, Kermanshah, Iran; ^7^ Department of Epidemiology, School of Public Health, Iran University of Medical Sciences, Tehran, Iran; ^8^ Probiotic Research Center, Alborz University of Medical Sciences, Karaj, Iran; ^9^ Social Determinants of Health Research Center, Alborz University of Medical Sciences, Karaj, Iran; ^10^ Chronic Diseases Research Center, Endocrinology and Metabolism Population Sciences Institute, Tehran University of Medical Sciences, Tehran, Iran

**Keywords:** dyslipidemia, non-high-density lipoprotein cholesterol, metabolic syndrome, cardiovascular disease, cardiometabolic, cholesterol

## Abstract

**Introduction:**

Novel atherogenic lipid indices, including non-high-density lipoprotein cholesterol (non-HDL-C) which is calculated by subtracting the HDL-C value from the total cholesterol level, atherogenic index (ratio between triglycerides (TG) and HDL-C concentrations (TG/HDL-C)), and Diff-C (calculated by subtracting low-density lipoprotein (LDL-C) from non-HDL-C), have been known as valuable predictors of dyslipidemia and subsequent cardiovascular diseases. Previous studies have reported the potential association of novel atherogenic lipid indices with metabolic syndrome (MetS). This meta-analysis aimed to assess the pooled association of novel atherogenic lipid indices with MetS or its components.

**Methods:**

A systematic search was conducted through PubMed, Scopus, and Web of Science (WoS) databases from January 2000 until March 2021 to evaluate the association of novel atherogenic lipid indices, including non-HDL-C, atherogenic index, and the difference between non-HDL-C and LDL-C (Diff-C) with MetS. Observational studies were included without any language restriction. As exclusive studies evaluating the association of non-HDL-C with metabolic syndrome (MetS) were eligible to be included in quantitative analyses, a random-effect meta-analysis was performed to pool the odds ratios (ORs). A stratified meta-analysis was performed based on the definition of MetS [Adult Treatment Panel (ATP) and International Diabetes Federation (IDF)] and the studied population.

**Results:**

Overall, 318 studies were retrieved from an initial systematic search. After screening, 18 and five studies were included in the qualitative and quantitative syntheses, respectively. Qualitative synthesis revealed an association between non-HDL-C, Diff-C, and atherogenic index with MetS and its components. Stratified meta-analysis showed that an increased non-HDL-C level was associated with an increased odds of MetS based on ATP criteria (OR: 3.77, 95% CI: 2.14-5.39) and IDF criteria (OR: 2.71, 95% CI: 1.98-3.44) in adults (OR: 3.53, 95% CI: 2.29-4.78) and in children (OR: 2.27, 95% CI: 1.65-2.90).

**Conclusion:**

Novel atherogenic lipid indices, including atherogenic index, Diff-c, and non-HDL-C, are strongly associated with increased odds of MetS and its components. The indices could be considered as potential predictors of MetS and its components in clinical practice.

## Introduction

Metabolic syndrome (MetS) is a well-established risk factor which increases the likelihood of experiencing cardiovascular events (1). The current study considers diagnoses of metabolic syndrome (MetS) and other cardiometabolic risk factors such as hypertension, central obesity, insulin resistance, hyperinsulinemia, diabetes, and hyperlipidemia as outcomes. It is estimated that the prevalence of these risk factors has risen remarkably (2, 3). Five of these risk factors comprise a syndrome called MetS. Although several definitions of MetS have been introduced, the five parameters serum glucose levels, high-density lipoprotein cholesterol (HDL-C), triglyceride (TG), obesity, and blood pressure have generally been the defining factors of the syndrome (4). Among all MetS components, hyperlipidemia has been recognized as an independent and significant risk factor for cardiovascular disease (CVD) (5). According to the Framingham Heart Study, among the parameters measured in the lipid profile, a low level of HDL-C and a high level of low-density lipoprotein cholesterol (LDL-C) are strongly associated with the increased risk of CVDs (6). This information reveals that the incident risk of CVD is increased by 2%–3% with each mg/dL decrease in HDL-C levels (7). The Framingham Heart Study’s findings on LDL-C have been repeatedly confirmed by other studies (8–10) to the point that controlling LDL-C levels is currently recognized as the primary target in treating hyperlipidemia (11).

Based on the National Cholesterol Education Program Adult Treatment Panel (NCEP ATP III) suggestion, the secondary target in treating hyperlipidemia in patients with a triglyceride higher than 200 is non-high-density lipoprotein cholesterol (non-HDL-C) (11). Non-HDL-C measures LDL-C, VLDL-C, chylomicrons, lipoprotein(a), IDL, and chylomicron remnant. Non-HDL cholesterol (non-HDL-C) is calculated by subtracting the HDL-C value from the total cholesterol level. Although several components make up non-HDL, this index mainly comprises atherogenic lipoproteins such as LDL, very low-density lipoprotein (VLDL-C), and intermediate-density lipoprotein (IDL-C). Different studies have shown that even after a significant decrease in LDL-C levels, a considerable amount of residual risk for CVD incidence remains. It was concluded that other lipids (other than LDL-C) are also involved in increasing the risk of CVD (12, 13). One of the indices measuring these lipids is non-HDL-C. Non-HDL-C measures different components such as LDL-C, VLDL-C, chylomicrons, lipoprotein(a), IDL, and chylomicron remnants. Moreover, the atherogenic index (ratio between TG and HDL-C concentrations (TG/HDL-C) and Diff-C (calculated by subtracting LDL-C from non-HDL-C)) measures the cumulative effects of these lipids on the CVD risk increment. The data extracted from Framingham’s study show that some of these components, such as VLDL-C, even further increase the risk of CVD incidence compared to LDL-C; the importance of this result is so significant that a study demonstrated that after multivariate adjustment for the non-HDL-C level, LDL-C would not increase the risk of CVD independently (14).

The accompanying of high non-HDL-C and other metabolic syndrome parameters showed a cumulative increment in CVD mortality risk. In other words, the risk of developing CVD is 200 times higher in diabetic patients than in non-diabetic patients (15). If diabetes is accompanied by dyslipidemia, the risk of CVD is further increased in the patients. Prior studies have searched for lipid targets to help decrease this added risk (16–18), and they conclude that compared to LDL-C, non-HDL-C is a stronger predictor for CVD fatality in diabetic patients (19). This study aims to evaluate the association of non-HDL-C, atherogenic index, and Diff-C with MetS and its components.

## Methods

This systematic review and meta-analysis were performed according to the Preferred Reporting Items for Systematic Reviews and Meta-Analyses (PRISMA) statement.

### Study question

• Are novel atherogenic lipid indices associated with metabolic syndrome?

### Information sources and search strategy

A systematic search was independently carried out through PubMed, Scopus, and Web of Science (WoS) databases (from January 2000 until March 2021) by two reviewers (ES and FA) on the link of MetS and the atherogenic index, Diff-c, or non-HDL-C. The search strategy is demonstrated in **Supplementary Table 1**. Moreover, other resources, related gray literature, publications’ reference lists, and related key journals were searched for additional publications.

### Study selection

EndNote reference management software was used for the study selection process so as to manage the papers. After removing duplicate papers, the title and abstract of the articles were evaluated based on the inclusion criteria. Eventually, the full texts were screened in detail. The selection process was independently conducted by two authors (PM and MQ).

### Eligibility criteria

The following criteria were considered for screening the included articles: 1) observational studies which include participants’ novel atherogenic lipid indices including atherogenic index, Diff-C, or non-HDL-C level; 2) articles must include data on patients’ MetS or its components’ diagnosis, including hypertension, obesity, insulin resistance, hyperinsulinemia, diabetes, hyperlipidemia, and coronary heart disease; 3) articles must demonstrate a link between MetS or its components’ diagnosis and the atherogenic lipid indices; 4) articles can be published in any language.

### Data collection process and data items

The data extraction form has been filled by two researchers independently. Another researcher resolved conflicts.

### Quality assessment

Quality assessment was conducted by the Strengthening the Reporting of Observational Studies in Epidemiology (STROBE) Statement. This statement provides general reporting recommendations for descriptive observational studies and studies which investigate the associations between exposures and health outcomes. Both of these guidelines consist of 25 subitems. Each of these subitems was rated yes (1 point) or no (0 points); the final quality assessment score is the sum of these subitem points. The quality assessment was carried out by two researchers independently based on the guidelines’ items.

### Data synthesis

Results are presented as odds ratio (OR) and its 95% confidence interval (95% CI). STATA version 11.2 (StataCorp, College Station, TX) software was used to conduct the meta-analysis. We conducted a meta-analysis when two or more than two studies report the association between an atherogenic lipid index with MetS or its components. The pooled estimate of ORs and their 95%CI were calculated based on extracted data from the studies which were included in quantitative analysis. The heterogeneity was evaluated based on the I^2^ statistic and the chi-square-based Q test. Lack of heterogeneity was defined when the p-value was more than 0.10. Random or fixed effect models were used to pool the association of non-HDL-C-C with MetS. Subgroup analysis was used based on the study population (adults/children) and criteria (ATP III/IDF). Publication bias was assessed by using Begg’s test. We considered a substantial publication bias whenever the p-value was calculated less than 0.1. Sensitivity analysis was performed to assess the effect of exclusion of studies which did not adjust the potential confounders.

## Results

### Study and patient characteristics

**Figure 1:**
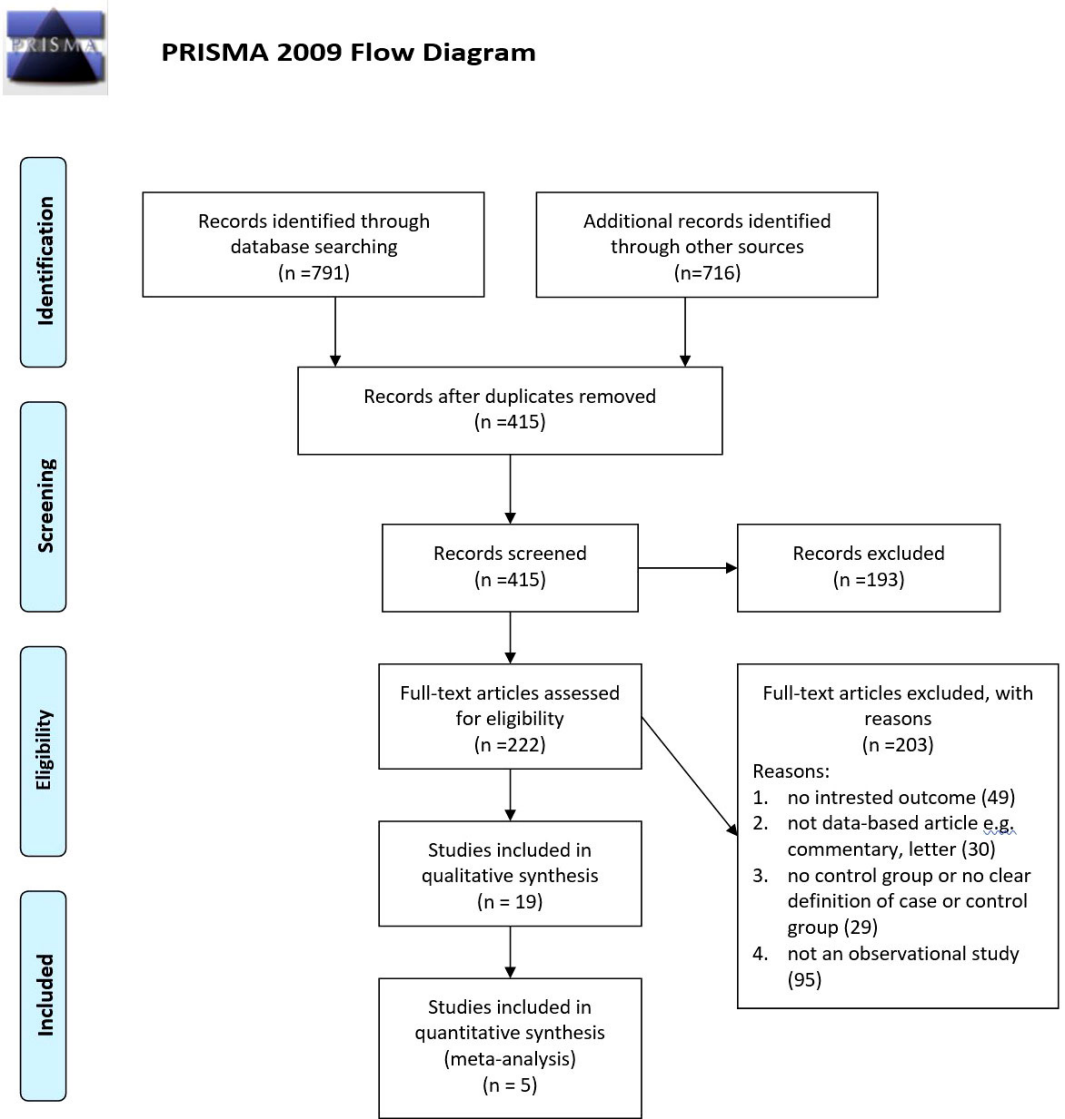
PRISMA diagram for searching resources.

Our searches revealed 269 studies from PubMed, 317 studies from Scopus, and 205 studies from the Web of Science. In addition, our manual search for gray literature yielded 456 studies. After the rejection of duplicates, we screened 415 studies, followed by a full-text assessment for eligibility for 222 papers. Finally, 19 (20–38) and 5 (20, 25, 27, 29, 31) studies were included in the qualitative and quantitative syntheses, respectively. The detailed flow diagram is demonstrated in **Figure 1**. Four of the included studies were cohorts, while 14 of them were cross-sectional studies. Seven studies were originated from the United States, followed by three papers that originated from Iran. The largest sample size was for Miyazaki et al.’s study with 5,853 participants, and the smallest sample size was for Dharuni et al.’s study with 100 participants. Studies’ provenance, sample size, target population, and their patients’ characteristics are summarized in **Table 1**.

**Table 1:** Characteristics of the included studies.

Author (ref)	Year	Study type	Country	Target population	Sample size	Sex ratio (M/F)	Age (year)	Quality score
Angoorani (20)	2018	Cross-sectional	Iran	Healthy children and adolescents	3,843	2,010/1,833	7-18	22*
Dharuni (21)	2016	Cross-sectional	India	Metabolic syndrome	100	35/65	Case: 50.4 ± 9.7 Control: 50.2 ± 9	20*
Frontini (23)	2007	Cohort	USA	Asymptomatic younger adult	1,203	43% man, 71% white	24-34	18**
Frontini (22)	2008	Cohort	USA	Children	437	40% male, 70% white	5-19	19**
Gasevic (24)	2014	Cross-sectional	Aboriginal, Chinese, European, and South Asian origin	Healthy adult	797	380/417	35-60	20*
Ghodsi (25)	2017	Cross-sectional	Iran	Adults	2,125	957/1,168	25-64	21*
Huang (26)	2008	Cross-sectional	United States	Adults	928	297/631	53	21*
Kazemi (27)	2010	Cross-sectional	Iran	Healthy adults	3,277	1,578/1,699	15<	16*
Khan (28)	2018	Cross-sectional	Pakistan	Asymptomatic subjects referred for CVD risk evaluation	229	109/120	Male:47.98 ± 11.30 females: 45.27 ± 12.42	19*
Lee (29)	2007	Cross-sectional	Korean	Women	511	–	48.36 ± 5.29	18*
Li (30)	2008	Cross-sectional	US	Non-diabetic adults	2,652	1,358/1,294	≥20	17*
Li (31)	2011	Cross-sectional	US	Healthy children and adolescents	2,734	1,444/1,290	12-19	20*
Liang (32)	2015	Cross-sectional	China	Obese children	976	690/286	6-16	19*
Liu (33)	2013	Cross-sectional	US	Healthy adult	366	143/223	22-70	19*
Miyazaki (34)	2016	Cross-sectional	Japan	schoolchildren	5,853	2,963/2,890	6-15	20*
Onat (35)	2010	Cohort	Turkey	Middle-age adult 7.8-year follow-up	2,676	1,294/1,382	28-80	21**
Park (36)	2015	Cross-sectional	Korea	Adult males who visited the Health Promotion Center and underwent medical examination and abdominal CT	372	1	Mean: 52	18*
Srinivasan (37)	2002	Cross-sectional	US	Healthy children	2,843	1,422/1,421	5-17	20*
Srinivasan (38)	2006	Cohort	US	Healthy children	1,163	519/644	Children: 5-14-year adults: 27<	19**

*Quality assessed by STROBE for cross-sectional studies. **Quality assessed by STROBE for cohort studies.

### Qualitative synthesis

#### Diagnostic values of Diff-C, non-HDL-C, and atherogenic index

Ten of included papers reported diagnostic values, including sensitivity, specificity, the area under the ROC curve (AUC) of Diff-C, non-HDL-C, and atherogenic index to predict metabolic syndrome or one of its diagnostic components. The highest sensitivity, specificity, and AUC for Diff-C reported in the literature were 0.86 (0.78-0.93) (20), 89.1 (25), and 0.828 (0.770-0.887) (25), respectively. Similarly, non-HDL-C showed a sensitivity ranging from 0.22 in Liu et al.’s (33) study to predict insulin resistance to 75.7 in Ghodsi et al.’s (25) study to predict MetS diagnoses by ATP III criteria. While the highest specificity for non-HDL-C was 89% for patients diagnosed with MetS by Harmonious criteria (33), the lowest specificity was reported 57.1 in Ghodsi et al.’s (25) study for patients diagnosed with MetS IDF criteria. Likewise, the atherogenic index showed AUC, ranging from 0.625 (32) to 0.872 (24). **Table 2** demonstrates diagnostic values of Diff-C, non-HDL-C, and atherogenic index to predict MetS or its components.

**Table 2:** Characteristics of the included studies which assessed the diagnostic value of Diff-C, non-HDL, and atherogenic index to predict CMRFs.

Author, year	Outcome	Diagnostic criteria	Cutoff value		SE %(95% CI)	SP %(95% CI)	AUC (95% CI)
Diff-C (mg/dL)							
Angoorani, 2018 (20)	MetS	ATP III for pediatrics	M	19.9 (19.26-20.33)	0.84 (0.76-0.91)	0.76 (0.73-0.79)	0.80
			F	19.9 (19.37-20.22)	0.86 (0.78-0.93)	0.74 (0.70-0.78)	0.80
Ghodsi, 2017 (25)	Mets	ATP III	29.55		73.3	82.9	0.819 (0.801,0.838)
		ATP III in DM (−)	30		72.4	88.3	0.817 (0.797, 0.834)
		ATP III in DM (+)	30		70.3	89.1	0.828 (0.770, 0.887)
		IDF	29.50		65.9	80.4	0.777 (0.757, 0.797)
		IDF in DM (−)	29.45		67.5	79.6	0.786 (0.765, 0.807)
		IDF in DM (+)	30		68.2	59	0.627 (0.549, 0.705)
Non-HDL-C							
Angoorani, 2018 (20)	MetS	ATP III for pediatrics	M	119.5 (103.37,134.62)	0.49(0.26-0.71)	0.73 (0.50-0.95)	0.61
			F	115.5(88.58,141.4)	0.49 (0.18-0.78)	0.64 (0.25-1.01)	0.56
Liu, 2013 (33)	MetS	“Harmonious” criteria	160		0.46	0.72	–
			190		0.24	0.89	–
	Insulin resistance	SSPG ≥10.3 mmol/l	160		0.44	0.69	–
			190		0.22	0.87	–
Li, 2011 (31)	MetS	ATP III for pediatrics	120		0.75	0.69	0.77 (0.73-0.81)
		ATPIII for adults	120		0.73	0.75	0.81 (0.76-0.86)
		IDF for pediatric	120		0.67	0.75	0.79 (0.74-0.84)
		IDF for adult	125		0.68	0.75	0.78 (0.73-0.83)
Ghodsi,2017 (25)	Mets	ATP III	153.5		0.75	0.57.2	0.719 (0.697, 0.740)
		ATP III in DM (−)	161.5		0.67	0.64.1	0.717 (0.693, 0.740)
		ATP III in DM (+)	175.5		0.55	0.84.8	0.733 (0.659, 0.807)
		IDF	153.5		0.73	0.57.1	0.693 (0.670, 0.715)
		IDF in DM (−)	160		0.67	0.63.4	0.698 (0.674, 0.722)
		IDF in DM (+)	175.8		0.54	0.65.3	0.608 (0.534, 0.683)
Frontini, 2008 (22)	Excess carotid IMT in children	Top 10th percentile	–		–	–	0.65 (0.56-0.70)
Frontini, 2007 (23)	Increased carotid intima-media thickness in adults	Top 10th percentile	–		–	–	0.73 (0.68-0.78)
Miyazaki, 2016 (34)	Cardiovascular disease/MetS	Takaoka/nationwide	152 mg/dL (97th percentile)		0.98	–	–
Atherogenic index							
Angoorani,2018 (20)	MetS	ATP III for pediatrics	M	2.53 (2.35,2.71)	0.80 (0.71-0.88)	0.80 (0.76-0.83)	0.80
			F	2.54 (2.19,2.89)	0.86 (0.77-0.94)	0.79 (0.71-0.86)	0.83
Gasevic, 2014 (24)	Mets	Number of Mets components	M	1.62	0.84	0.80	0.869 (0.830, 0.908)
			F	1.18	0.70	0.88	0.872 (0.832, 0.912)
Li, 2008 (22)	Hyperinsulinemia	FSI of 13.13 µU/ml (the 75th percentile)	NHW	1.2	0.70	0.71	0.77 (0.74 to 0.79)
			NHB	0.9	0.61	0.77	0.75 (0.69 to 0.77)
			MA	1.2	0.64	0.71	0.74 (0.69 to 0.76)
Liang, 2015 (32)	Mets	MS-CHN2012	1.25		0.80	0.75	0.843
	Insulin resistance	HOMA1-IR	4.59		0.59	0.66	0.640
		HOMA2-IR	2.76		0.53	0.70	0.625

MetS, metabolic syndrome; ATP III, Adult Treatment Panel III; IDF, International Diabetes Federation; M, male; F, female; MA, Mexican American; NHW, non-Hispanic white; NHB, non-Hispanic black.

#### Association of Diff-C, non-HDL-C, and atherogenic index and MetS or its components

Our search yielded 14 articles measuring OR, correlation coefficient, risk ratio, Spearman correlation, Pearson correlation, multiple linear regression, t-test, and Poisson regression analysis to evaluate the association of Diff-C, non-HDL-C, and atherogenic index with MetS or its components. Regarding the association of MetS and Diff-C, the highest adjusted OR was 26.29 (17.71-39.05) in patients diagnosed by ATP III, followed by 10.71 (7.47–15.35) in patients diagnosed by IDF, both reported in Ghodsi et al.’s study (25). Non-HDL-C showed a relatively strong correlation with MetS with ORs as high as 5.87 (3.92-8.80) (25) and Spearman correlation results as high as 0.95 p < 0.0001 (37). Similarly, ORs reported for atherogenic index and MetS range from 1.00 (0.92 to 1.09) in Angoorani et al.’s (20) study per one-unit increment of the atherogenic index to predict high blood pressure to 40.26 to predict high triglyceride level (20) (**Table 3**).

**Table 3:** Characteristics of the included studies which assessed relationship between Diff-C, non-HDL, and atherogenic index and CMRFs.

Author, year	Outcome	Definition of outcome	Cutoff for Diff-C, non HDL, and atherogenic index	Type of effect size	effect size			Confounder
Diff-C								
Angoorani, 2018 (20)	High TC (mg/dl)	More than 200	Per 1-mg/dl increment.	Adjusted odds ratio (95 % CI)	1.07(1.06-1.09)*			Adjusted for age, sex, living area, screen time, SES and physical activity and adjusted for BMI except for overweight, obesity and abdominal obesity.
	High LDL(mg/dl)	More than 110			1.02(1.01-1.03)*			
	MetS	ATP III			1.08(1.07-1.10)*			
	Low HDL (mg/dl)	Less than 40 mg/dl, except for boys between 15 and 19 years old; which is less than 45 mg/dl			1.04(1.04,1.05)*			
	Overweight (Kg/m2)	85th < BMI < 95th			1.01 (1.00-1.02)*			
	Abdominal Obesity	Waist to height ratio more than 0.5			1.00 (0.99-1.01)			
	Obesity (Kg/m2)	BMI more than 95th			1.00 (0.99-1.01)			
	High FBS (mg/dl)	More than 100			1.03 (1.02-1.05)*			
	High TG (mg/dl)	More than 100			1.02 (1.01-1.03)*			
	Hypertension (mmHg)	More than 90th			1.00 (0.98-1.01)			
Ghodsi, 2017 (25)	Mets (IDF)	ATP III	30 mg/dl	Adjusted odds ratio (95% CI)	26.29 (17.71- 39.05)			Age, sex, residential area, Hypertension, total physical activity, waist circumference, FBS, Insulin resistance (HOMA.IR), and BMI
		IDF			10.71 (7.47-15.35)			
Non-HDL-C								
Angoorani, 2018 (25)	High TC(mg/dl)	More than 200 mg/dl	Per 1-mg/dl increment	Adjusted odds ratio (95% CI)	1.19 (1.16,1.22)*			Age, sex, living area, screen time, SES and physical activity; additionally for BMI except for BMI, and WC outcomes.
	High LDL (mg/dl)	More than 110			1.19 (1.17,1.21)*			
	MetS	ATP III			1.01 (1.00, 1.01)			
	Low HDL (mg/dl)	Less than 40 mg/dl, except for boys between 15 and 19 years old; which is less than 45 mg/dl			0.99(0.99,0.99)			
	Overweight (Kg/m2)	85th < BMI < 95th			1.00 (0.99,1.00)			
	Abdominal Obesity	Waist to height ratio more than 0.5			1.00(0.99,1.00)			
	Obesity (Kg/m2)	BMI more than 95th			1.00(0.99,1.00)			
	High FBS (mg/dl)	More than 100 mg/dl			1.00(.99,1.01)			
	High TG (mg/dl)	More than 100			1.03(1.02,1.03)*			
	High BP (mmHg)	More than 90th percentile			0.99 (0.99,1.01)			
Huang, 2008(26)	MetS	Diagnosed with MetS by ATP III	Reporting non-HDL value in each group	T-test (mean ± SD)	M:174±64			None
					F:165±50			
		Not diagnosed with MetS by ATP III			M:156±57			
					F:147±41			
Liu, 2013 (33)	Waist circumference (cm)	As a continuous variable	As a continuous variable	Correlation coefficient (r)	0.25*			Age, sex, BMI
	SBP (mmHg)				0.24*			
	DBP (mmHg)				0.21*			
	FBS (mg/dl)				0.13			
	HDL(mg/dl)				−0.19*			
	TG (mg/dl)				0.46*			
Li, 2011 (31)	MetS	ATP III for pediatrics	120 mg/dl	Adjusted odds ratio (95% CI)	2.8 (1.7-4.8)*			Sex, age, race/ethnicity, and poverty-to-income ratio, cotinine, C-reactive protein, fasting insulin, BMI
			145 mg/dl		4.0 (2.4-6.9)*			
		ATP III for adult	120 mg/dl		3.5 (1.8-6.9)*			
			145 mg/dl		5.6 (2.6-12.3)*			
		IDF for pediatric	120 mg/dl		3.2 (1.6-6.5)*			
			145 mg/dl		4.5 (2.1-9.6)*			
		IDF for adult	120 mg/dl		3.0 (1.6-5.6)*			
			145 mg/dl		3.9 (1.9-7.9)*			
Srinivasan, 2006 (38)	Dyslipidemia	Receiving medication for dyslipidemia	More than144 mg/dl versus less than 123 mg/dl	Adjusted odds ratio (95% CI)	4.49 (2.51 – 8.04)*			Baseline BMI and change after 27 years.
	Obesity	BMI greater than or equal to 30 kg/m2		Prevalence odds ratio (95% CI)	1.9438 (1.0866 - 3.4773)*		
	High LDL(mg/dl)	LDL greater than or equal to 160			4.6885 (2.2713 - 9.6782)*		
	High TG(mg/dl)	TG greater than or equal to 150			3.1441 (1.7000 - 5.8148)*		
	Low HDL(mg/dl)	HDL less than 40			1.8387 (1.0025 - 3.3725)*		
	High FBS(mg/dl)	FPG greater than or equal to 126			2.8116 (0.7236 - 10.9243)		
	High Insulin (µU/mL)	Insulin more than 18			1.8446 (0.9190 - 3.7026)		
	Hypertension	SBP more than 140 mm Hg in addition to DBP more than 90 mm Hg			1.8434 (0.7989 - 4.2534)		
Ghodsi, 2017 (25)	Mets	ATP III	160 mg/dl	Adjusted odds ratio (95% CI)	2.75 (2.10, 3.61)*			Age, sex, residential area, hypertension, total physical activity, waist circumference, FBS, Insulin resistance (HOMA.IR), and BMI
			190 mg/dl		3.61 (2.67, 4.88)*			
			Q2 versus Q1		1.78(1.18, 2.70)*			
			Q3 versus Q1		2.62(1.74, 3.95)*			
			Q4 versus. Q1		5.87(3.92, 8.80)*			
		IDF	160 mg/dl		3.14(2.30, 4.29)*			
			190 mg/dl		2.70(2.03, 3.59)*			
			Q2 versus Q1		1.43(0.85, 2.44)			
			Q3 versus Q1		3.08(1.83, 5.19)*			
			Q4 versus. Q1		4.90(3.00, 8.16)*			
Srinivasan,2002 (27)	BMI(Kg/m2)	As a continuous variable	As a continuous variable	Spearman correlation	0.13*			Age, race, gender, cigarettes/week, and alcohol (mL/week).
	WC (cm)				0.09*			
	TC(mg/dl)				0.9*			
	TG(mg/dl)				0.42*			
	LDL (mg/dl)				0.95*			
	HDL (mg/dl)				-0.12*			
Onat, 2010 (35)	Diabetes	AHA criteria	Per 40-mg/dl increment	Risk ratio (95% CI)	M;1.27 (1.00–1.60)			Age, BP, smoking, BMI, atherogenic index
					F; 1.13(0.85–1.49)			
	Coronary heart disease	The presence of angina pectoris, of a history of myocardial infarction with or without accompanying Minnesota codes of the electrocardiogram			M; 1.49 (1.22–1.81)*			
					F; 1. 32(1.04–1.61)*			
Lee, 2007 (39)	Mets	ATP III	T3 vs. T1	Adjusted odds ratio (95% CI)	4.005 (1.151-13.939)*			BMI, age, BP,FBS, atherogenic index
		IDF	T3 vs. T1		1.772 (0.510-6.161)			
Khan, 2018 (28)	BMI (Kg/m2)	As a continuous variable	As a continuous variable	Pearson correlation (r)	0.139*			BMI, age, BP, WHpR, fasting plasma glucose, A1c, insulin, HOMA-IR, urine albumin creatinine ratio
	SBP (mmHg)				0.078			
	DBP (mmHg)				0.110			
	WHpR				0.191*			
	FBS(mg/dl)				0.071			
	HbA1c (mg/dl)				-0.040			
	Insulin				0.109			
	HOMA-IR				0.125			
Kazemi, 2010 (27)	Mets	ATP III	190 mg/dl	Adjusted odds ratio (95% CI)	5.1 (4.1-6.2)*			BMI, waist circumstance, BP,LDL, cholesterol, triglycerides, HDL-C, VLDL, LDL, non-HDL-C,HDL-C
Atherogenic index								
Angoorani, 2018(20)	High TC (mg/dL)	More than 200	Per 1 increment	Adjusted odds ratio (95% CI)	1.35 (1.24,1.47)			age, sex, living area, screen time, SES and physical activity; additionally for BMI except for BMI and WC outcomes.
	High LDL (mg/dL)	More than 110			1.03 (0.96,1.10)			
	MetS	ATP III			1.9(1.80- 2.19)*			
	Low HDL (mg/dl)	Less than 40 mg/dl, except for boys between 15 and 19 years old; which is less than 45 mg/dl			2.50(2.30-2.72)*			
	Overweight (kg/m2)	85th < BMI < 95th			1.07(0.98-1.15)			
	Abdominal obesity	Waist-to-height ratio more than 0.5			1.01(0.95-1.08)			
	Obesity (kg/m2)	BMI more than 95th percentile			1.03(0.95-1.12)			
	High FBS (mg/dL)	More than 100 mg/dl			1.28 (1.18-1.40)*			
	High TG (mg/dL)	More than 100			40.26(30.36-53.40)*			
	High BP(mg/dL)	More than 90th			1.00 (0.92-1.09)			
Li, 2008 (30)	Fasting serum insulin	As a continuous variable	As a continuous variable	Multiple linear regression βm (SE)	Men, NHW; 0.19 (0.02)			age, education attainment, poverty-income ratio, smoking, systolic blood pressure, C-reactive protein, and waist circumference
					M, NHB; 0.24 (0.04)			
					M, MA; 0.22 (0.04)			
					F, NHW; 0.24 (0.05)			
					F, NHB: 0.21 (0.05)			
					F, MA: 0.34(0.03)			
	Hyperinsulinemia	More than 78.77 pmol/l (or 13.131 µU/ml)	3.5	Prevalence ratio (95% CI)	NHW: 2.3(1.7-3.1)*			
			3.0		NHW: 2.3(1.8 – 3.0)*			
			3.5		NHB: 1.9(1.5 – 2.5)*			
			2.0		NHB: 2.1(1.5-2.9)*			
			3.5		MA: 1.8(1.5 – 2.2)*			
			3.0		MA: 2.0(1.6 – 2.5)*			
Onat, 2010(35)	Fasting insulin	Per 1 mIU/l	As a continuous variable	Spearman correlation results in first column and multiple linear regression results in second column β (SE)	M: 0.28*		1.26 (1.11)	age, BP, Smoking, BMI, atherogenic
					F: 0.20*		1.02 (1.10)	
	BMI (kg/m2)	Per 5 kg/m2			M: 0.34*		1.08 (0.02)	
					F: 0.29*		1.04 (0.01)	
	Waist circumference (cm),	Per 11 cm			M: 0.32*			
					F: 0.29*			
	TC (mg/dL)	Per 1.03-mmol/l increment			M: 0.32*		1.15 (0.04)	
					F: 0.31*		1.07 (0.04)	
	LDL-cholesterol (mg/dL)	Per 0.93-mmol/l increment			M: 0.12*		0.90 (0.04)	
					F: 0.22*		0.96 (0.04)	
	FBS (mg/dL)	Per 1.39-mmol/l increment			M: 0.06*		1.05 (0.008)	
					F: 0.11*		1.03 (0.008)	
	SBP (mmHg)	Per 25-mmHg increment			M: 0.11*		1.004 (0.025)	
					F: 0.20*		1.016 (0.025)	
	DBP (mmHg)	Per 25-mmHg increment			M: 0.16*			
					F: 0.19*			
	Hypertensio n	140,90	Q4 versus Q1 (Q4 for men = 2.26 woman = 2.99 and Q1 = 1 for both genders)	Risk ratio (95% CI)	M: 1.35 (0.87–2.09)			systolic BP, smoking status, BMI, and total and LDL-cholesterol

					F: 1.47(0.94–2.29)			
	Diabetes	AHA	Per 0.3 increment		M:1.15 (0.90–1.47)			
					F: 1.09 (0.83–1.44)			
	MetS	ATP III	Q4 versus Q1 (Q4 for men = 2.26 woman = 2.99 and Q1 = 1 for both genders)		M: 7.81 (3.90–15.6)*			
					F: 6.72 (3.22–14.0)*			
	Coronary heart disease	The presence of angina pectoris, of a history of myocardial infarction with or without accompanying Minnesota codes of the electrocardiogram	Per 0.3 increment		M: 1.28 ( 1.05 -1.57)*			
					F: 1.26 ( 1.01–1.56)*		
Gasevic, 2014(24)	Number of Mets components	As a continuous variable	As a continuous variable	Poisson regression analyses	M:1.26 (1.19, 1.33)*			age, ethnicity, smoking, alcohol consumption, physical activity, family history of cardiovascular disease, BMI.for women: all + menopause status
					F:1.29 (1.20, 1.36)*			
Park, 2015(36)	BMI (kg/m2)	As a continuous variable	As a continuous variable	Multiple linear regression β (95% CI)	0.440( 0.293–0.588)*			age, smoking behavior, the frequency of alcohol intake/wk, and the frequency of exercis- ing/wk.
	Waist circumference (cm)				0.951( 0.547–1.355)*			
	SBP (mmHg)				0.419( -0.207–1.045)			
	DBP (mmHg)				0.225( -0.215–0.664)			
	A1c (mg/dL)				0.100( 0.051–0.150)*			
	FBS (mg/dL)				2.849( 1.698–4.001)*			
	Subcutaneous fat				1.270( -2.100–4.639)			
	Visceral fat				0.048( 0.027–0.068)*			
	Visceral-subcutaneous fat ratio (based on CT scan findings)				0.048( 0.027–0.068)*			
	BMI (kg/m2)	Greater than or equal to 25	3.0	Adjusted odds ratio (95% CI)	5.566(2.759–11.187)*			
	Waist circumference (cm)	Greater than or equal to 90			2.723(1.393-5.321)*			
	Visceral fat	Greater than or equal to 100			2.584(1.493-4.472)*			
	Hypertension	SBP more than 140 mm Hg in addition to DBP more than 90 mm Hg			1.204(0.572-2.535)			
	Diabetes mellitus	NR			2.746(1.447-5.212)*			

MetS, metabolic syndrome; ATP III, Adult Treatment Panel III; IDF, International Diabetes Federation; SBP, systolic blood pressure; DBP, diastolic blood pressure; BP, blood pressure; TG, triglycerides; FBG, fasting blood glucose; HDL-C, high-density lipoprotein cholesterol; TC, total cholesterol; LDL-C, low-density lipoprotein cholesterol; BMI, body mass index; ICC, intraclass (within-observer) correlation coefficients; ICR, intraclass coefficients of reliability; F, female; M, male. Quartiles of non-HDL-c defined as: Q1: non-HDL-c <132, Q2: 132–160, Q3:160–188, Q4: non-HDL-c > 188; tertile CC, correlation coefficient; CR, coefficients of reliability; OR, odds ratio; POR, prevalence odds ratio; PC, Pearson correlation; RC, regression coefficients; SC, Spearman coefficient; *Statistically significant.

### Quantitative synthesis

**Figure 2:**
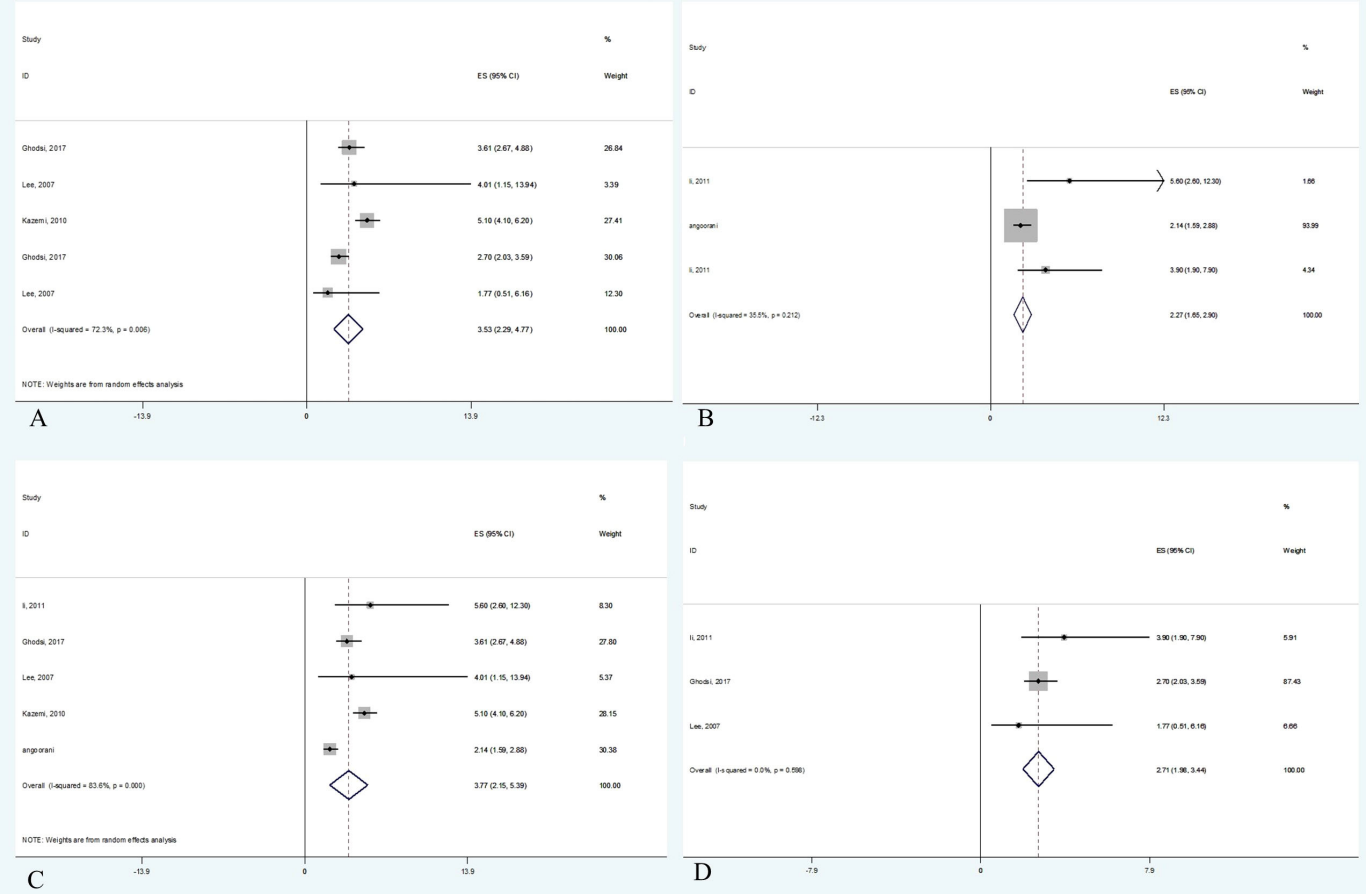
Forest plot of studies included in meta-analysis. **(A)** The association between non-HDL-C with metabolic syndrome in adults. **(B)** The association between non-HDL-C with metabolic syndrome in children. **(C)** The association between non-HDL-C with metabolic syndrome based on ATP III criteria. **(D)** The association between non-HDL-C with metabolic syndrome based on IDF criteria.

Data were from 17,860 participants of the five papers included in quantitative analysis which revealed that metabolic syndrome is linked with non-HDL-C in both adults (OR 3.53, 95% CI: 2.29-4.78) and children (OR 2.27, 95% CI: 1.65-2.90). Concerning the two different definitions used for metabolic syndrome in studies, the current meta-analysis demonstrated that the non-HDL-C level is correlated with metabolic syndrome using either ATP III diagnostic criteria (OR 3.77, 95% CI: 2.14-5.39) or IDF diagnostic criteria (OR 2.71, 95% CI: 1.98-3.44). The meta-analysis results are summarized in **Table 4**. Also, **Figure 2** illustrates the forest plot of included studies.

**Table 4:** Meta-analysis of the association between non-HDL-C with metabolic syndrome.

	Sample size	Pooled OR (CI)	Heterogeneity			
			Chi-square	I^2^	p-value	model
By study population						
Adults	8549	3.53 (2.29-4.78)	14.46	72.3	0.006	Random
Children	9311	2.27 (1.65-2.90)	3.10	35.5	0.212	Fixed
By MetS definition						
ATP III	12490	3.77 (2.14-5.39)	24.36	83.6	0.001	Random
IDF	5370	2.71 (1.98-3.44)	1.03	0.0	0.598	Fixed

MetS, metabolic syndrome; ATP III, Adult Treatment Panel III; IDF, The International Diabetes Federation.

#### Publication bias

**Figure 3:**
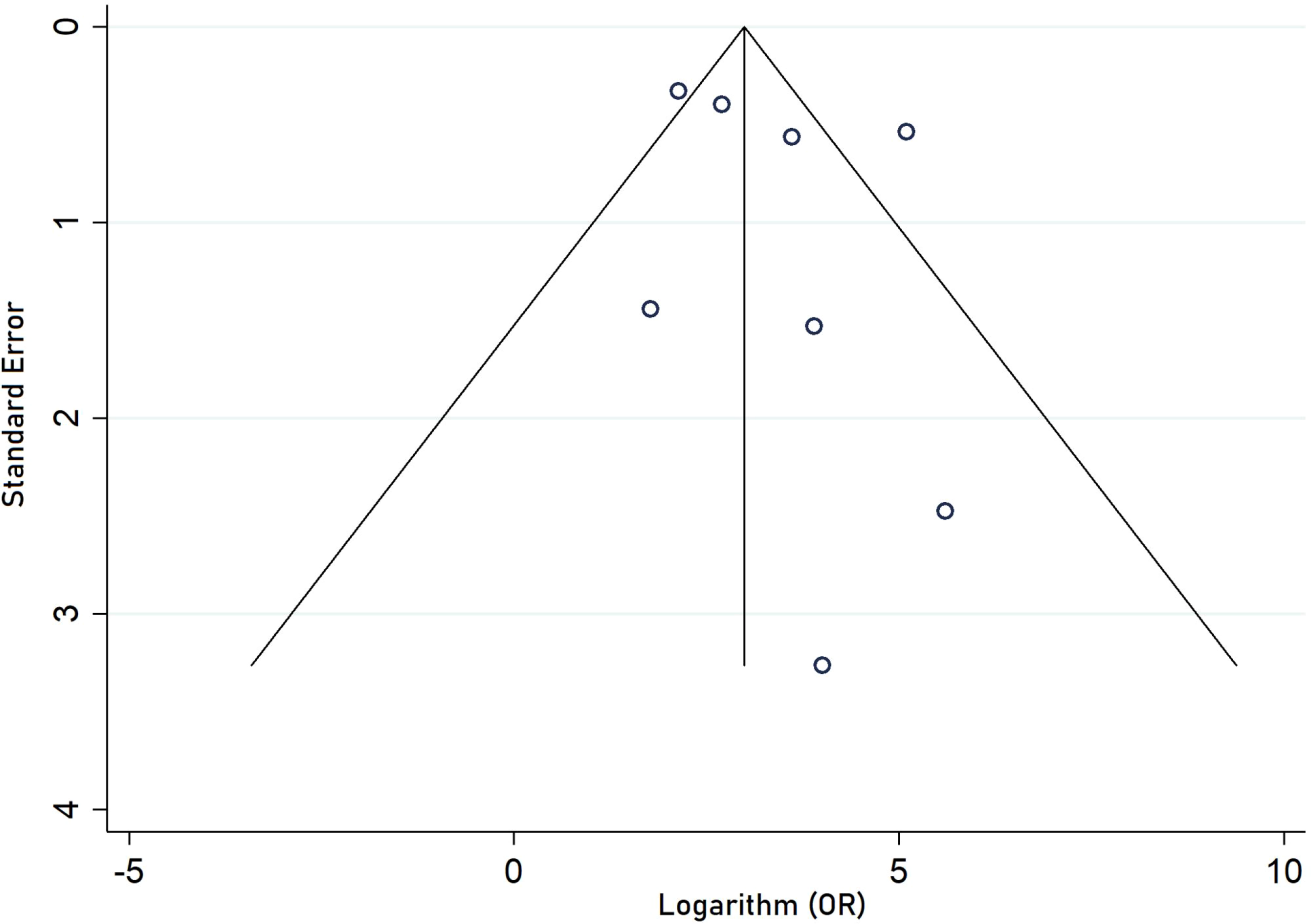
Funnel plot of studies included in quantitative analysis.

Begg’s (p = 0.567) showed no evidence of significant publication bias between non-HDL-C level and odds of being diagnosed with MetS. None of the included study population dramatically influenced the overall pooled OR. The funnel plot is demonstrated in **Figure 3**.

## Discussion

This study demonstrated that not merely can atherogenic lipid indices predict the diagnosis of MetS or its components but also these indices are correlated with higher odds of being diagnosed with these risk factors, including Mets, obesity, hypertriglyceridemia, reduced HDL cholesterol, diabetes, and hypertension.

In other words, our findings revealed that the odds of being diagnosed with MetS are nearly three times higher in patients with high non-HDL-C levels. Our data revealed that not only is non-HDL-C a reliable test to predict the MetS diagnosis in adults but also there are higher odds of being diagnosed with MetS in children with increased non-HDL-C levels. As pediatric MetS is a strong predictor of adulthood MetS (40), these patients are at a higher risk of type 2 diabetes and cardiovascular events (41). Moreover, the previous studies proved that interventions in pediatric MetS patients in early life could prevent MetS complications (42, 43). Therefore, this study proposes non-HDL-C as a marker to predict the odds of being diagnosed with MetS in pediatric patients.

In this study, we compared the criteria with which the MetS is diagnosed. It should be noted that in the same population the number of patients diagnosed by ATP III criteria is lower compared to patients diagnosed by IDF criteria (44). That is to say, ATP III has higher sensitivity, while IDF has higher specificity to diagnose MetS, and on average, patients diagnosed by ATP III are at a higher risk of cardiovascular events in comparison to patients diagnosed by IDF (45). Nevertheless, as confidence intervals regarding ATPIII and IDF overlap, this study demonstrated that the correlation of MetS and non-HDL is regardless of the MetS criteria.

Regardless of the criteria in which MetS is defined, it consists of five components, including obesity, hypertriglyceridemia, reduced HDL cholesterol, diabetes, and hypertension. The current study indicated a notable link between non-HDL-C and atherogenic index. Likewise, Sheth et al.’s study showed a significant correlation between obesity and non-HDL-C. Besides, they concluded that non-HDL-C and obesity have a cumulative role, and both should be considered possible biomarkers for CVD (46).

Another cardiometabolic risk factor is hypertriglyceridemia. This systematic review identified a notable association between hypertriglyceridemia and increased non-HDL-C and atherogenic index levels. Genetic and epidemiologic studies confirmed a causal association between elevated triglyceride and atherosclerosis (47, 48).

In the comparison of hypertriglyceridemia, and non-HDL-C, on the one hand, Puri et al. showed that non-HDL-C is more closely connected with coronary atheroma progression compared to triglyceride. In other words, non-HDL-C is linearly related to plaque progression, while only patients with triglyceride levels higher than 200 mg/dl showed an increment in risk of progression of coronary atheroma (49).

On the other hand, Bonito et al.’s study stated that non-HDL-C level is a weaker predictor for CVD incidence compared to triglyceride. It should be noted that their data demonstrated that patients with increased non-HDL-C and triglyceride levels are at a much higher risk of CVD compared to patients who solely have one increased parameter. That is to say, their data suggests that non-HDL-C and hypertriglyceridemia have a cumulative effect (50). Our data revealed that patients with increased non-HDL-C are at a higher risk of hypertriglyceridemia, and both are at a higher risk of MetS complications, especially CVD and atherosclerosis. For example, a cross-sectional article, which was conducted on 2,843 participants of the Bogalusa Heart Study, showed that non-HDL-C was related positively to triglycerides (Spearman correlation coefficient r = 0.42, p-value < 0.05) (37).

Another significant risk factor of CVD is diabetes. This study proved that diabetic patients have higher non-HDL-C levels compared to non-diabetic patients. Interestingly, diabetic patients with increased non-HDL-C levels are at a higher risk of severe coronary artery disease, regardless of their LDL-C level. In other words, in order to reduce CVD incidence in diabetic patients, not merely decreasing the LDL-C cholesterol is crucial but also reducing non-HDL-C levels should be considered (51).

This study showed that patients with increased non-HDL-C levels are at a higher risk of hypertension incidence. For example, Liu et al.’s article, a cross-sectional study conducted on 366 adult volunteers, showed that non-HDL-C levels and SBP and DBP are correlated (Spearman correlation coefficient r = 0.21, p-value < 0.05) (33). To justify this coincidence, Halperin et al. stated that dyslipidemia, especially high non-HDL-C level, is correlated with atherosclerosis, which may be an essential factor in the development of hypertension (52).

## Limitations

This study has some limitations which have to be addressed. First, the number of studies included was relatively low. In addition, some of these studies reported a small number of indices. Second, the heterogeneity of included studies, especially in terms of cutoff points of lipid, metabolic syndrome definition, and study population (children and adults), made the comparability of included articles challenging. Third, it should be noted that non-HDL comprises different lipoproteins, each of which affects the outcome differently. This study focused on overall effects of these lipoproteins, instead of assessing each of their effects, separately. Fourth, although we included adjusted studies in the meta-analysis, it should be considered that confounders may be different in included studies. Fifth, it should be considered that the Diff-C amount is alike the TG level; however, it also includes the remnant cholesterol (53).

## Conclusion

Although a limited number of studies were included in our study, non-HDL-C, Diff-C, and atherogenic index have shown to be associated with increased odds of being diagnosed with metabolic syndrome or its components. These findings were consistent in both adults and children and MetS diagnosed with both ATP III and IDF diagnostic criteria. Concerning the distinct designs and different diagnostic criteria, cohort studies with higher sample sizes should be conducted to more strongly evaluate the association between these lipid markers and MetS.

## Data availability statement

The original contributions presented in the study are included in the article/**Supplementary Material**. Further inquiries can be directed to the corresponding authors.

## Author contributions

PM interpreted the data and drafted the manuscript. MQ and AG designed the study, interpreted the data, reviewed the article critically, and revised it for important intellectual content. FA and AE participated in systematic search conduction. JH and ESh participated in data extraction, and quality assessment. PM, ES, and SD analyzed and interpreted the data. All authors contributed to the article and approved the submitted version.

## Funding

This study was funded by Alborz University of Medical Sciences.

## Conflict of interest

The authors declare that the research was conducted in the absence of any commercial or financial relationships that could be construed as a potential conflict of interest.

## Publisher’s note

All claims expressed in this article are solely those of the authors and do not necessarily represent those of their affiliated organizations, or those of the publisher, the editors and the reviewers. Any product that may be evaluated in this article, or claim that may be made by its manufacturer, is not guaranteed or endorsed by the publisher.
