# Supplementary material for: Is non-high-density lipoprotein associated with metabolic syndrome? A systematic review and meta-analysis
Source: Front Endocrinol (Lausanne). 2022 Sep 13;13:957136. doi: 10.3389/fendo.2022.957136 (PMC9514792; doi:10.3389/fendo.2022.957136)
Supplement: Supplementary file 1 [file Table_1.docx]

**Supplementary table**

| 1. | “Cardiometabolic Risk Factor” OR “Factor, Cardiometabolic Risk” OR “Factors, Cardiometabolic Risk” OR “Risk Factor, Cardiometabolic” OR “Risk Factors, Cardiometabolic” |
| --- | --- |
| 2. | “Non-HDL cholesterol” OR “non-HDL-C” OR “non hdl” OR “Atherogenic index” OR “Diff-C” |
| 3. | #1 AND #2 |
| Filters | Any language; Publication date (from January 2000, until March 2021); |
